# Effective synthesis of high-content fructooligosaccharides in engineered *Aspergillus niger*

**DOI:** 10.1186/s12934-024-02353-w

**Published:** 2024-03-09

**Authors:** Xiufen Wan, Lu Wang, Jingjing Chang, Jing Zhang, Zhiyun Zhang, Kewen Li, Guilian Sun, Caixia Liu, Yaohua Zhong

**Affiliations:** 1 State Key Laboratory of Microbial Technology, Institute of Microbial Technology, Shandong University, Qingdao, 266237 People’s Republic of China; 2 Shandong Academy of Pharmaceutical Sciences, Jinan, 250101 People’s Republic of China; 3 Baolingbao Biology Co., Ltd, Dezhou, 251299 People’s Republic of China

**Keywords:** Fructooligosaccharides, *Aspergillus niger*, Heterologous expression, β-fructofuranosidase, Glucose oxidase, Peroxidase

## Abstract

**Background:**

*Aspergillus niger* ATCC 20611 is an industrially important fructooligosaccharides (FOS) producer since it produces the β-fructofuranosidase with superior transglycosylation activity, which is responsible for the conversion of sucrose to FOS accompanied by the by-product (glucose) generation. This study aims to consume glucose to enhance the content of FOS by heterologously expressing glucose oxidase and peroxidase in engineered *A. niger*.

**Results:**

Glucose oxidase was successfully expressed and co-localized with β-fructofuranosidase in mycelia. These mycelia were applied to synthesis of FOS, which possessed an increased purity of 60.63% from 52.07%. Furthermore, peroxidase was expressed in *A. niger* and reached 7.70 U/g, which could remove the potential inhibitor of glucose oxidase to facilitate the FOS synthesis. Finally, the glucose oxidase-expressing strain and the peroxidase-expressing strain were jointly used to synthesize FOS, which content achieved 71.00%.

**Conclusions:**

This strategy allows for obtaining high-content FOS by the multiple enzymes expressed in the industrial fungus, avoiding additional purification processes used in the production of oligosaccharides. This study not only facilitated the high-purity FOS synthesis, but also demonstrated the potential of *A. niger* ATCC 20611 as an enzyme-producing cell factory.

**Supplementary Information:**

The online version contains supplementary material available at 10.1186/s12934-024-02353-w.

## Background

Fructooligosaccharides (FOS), one of the most important nondigestible oligosaccharides, have highlighted the importance in functional foods [1]. According to the Global Industry Analysts Inc. (GIA), there is a fantastic growth of FOS market in recent years and the global FOS market will reach $3.9 billion by 2027 [2]. It is known that FOS can selectively invigorate the growth of beneficial microorganisms in the colon, such as *bifidobacteria* and *lactobacilli* [3]. Naturally, FOS are found in plants items like onion, jerusalem artichoke, banana and some grasses [4]. FOS can be manufactured by extracting from plant materials, but the yield of FOS extracted from plants is relatively low and quite limited by the seasonal variation in plant abundance under natural conditions [4, 5]. Comparatively, the enzymatic conversion of sucrose to FOS provides a cost-effective and convenient method. In industry, FOS is generally synthesized from sucrose by the β-fructofuranosidase with transfructosylation activity at high sucrose concentrations [6]. The β-fructofuranosidase transfers the fructosyl group from the donor (sucrose) to the acceptor (sucrose or another FOS) to produce higher molecular weight FOS and simultaneously releases glucose [4]. Fructofuranosidases from *Aspergillus* sp. are widely used for the production of FOS from sucrose [7]. Especially, *A. niger* ATCC 20611 has been perceived as one of the most suitable industrial strains for FOS production due to its β-fructofuranosidase (FopA) possessing a high transfructosylation activity for sucrose-to-FOS biotransformation [8]. It has been reported that *A. niger* ATCC 20611 is capable of high-level production of FopA, which demonstrates much higher transfructosylation activity (U_t_) than hydrolysis activity (U_h_), with the U_t_/U_h_ ratio reaching up to 14.2 [9]. Besides the transfructosylation activity, the hydrolytic activity of FopA brings about glucose as by product, making the FOS mixtures with the content of approximately 55% [10]. However, the purity of FOS is important to promote physiological effects and place competitive advantage on the commercial market. For example, the FOS with over 90% of purity dominate the addition in the regular food/meal plan for all people with diabetes [11]. Hence, high-purity FOS need to be produced for the market demand [12].

Recently, developments in enzymology have facilitated enhancement of the FOS production using enzyme-aided optimization processes [13]. For example, Valdivieso-Ugarte et al. reported that supplementation of the extracellular glucose oxidase to the FOS synthesis reaction could remove the by-product glucose [14]. However, the use of free enzymes has some practical limitations such as low stability and high costs [15]. Furthermore, during the oxidation of glucose by glucose oxidase, the hydrogen peroxide is produced as by product and proved to have an inhibitory effect on the catalytic activity of glucose oxidase [16]. Consequently, the addition of peroxidase could contribute to the utilization of glucose, because the hydrogen peroxide could be immediately eliminated by peroxidases [17]. The combined system with glucose oxidase, glucose and peroxidase was reported to be used in the textile bleaching of natural cotton fibers and a significant higher degree of whiteness was obtained [17]. However, peroxidase and glucose oxidase have not been jointly used to enhance the FOS yield.

*Aspergillus niger* ATCC 20611 was used for the FOS synthesis in industry more than 30 years [18]. So far, optimization of the cultural and nutritional conditions of fermentation processes has been still the main method to optimize the process of enzyme production [5]. In addition, the fructofuranosidase activity of *A. niger* mycelia as the whole-cell biocatalyst substantially improved the simplicity of FOS production. More recently, Zhang et al. established an optimized polyethylene glycol (PEG)-mediated protoplast transformation system in *A. niger* ATCC 20611 and overexpressed FopA to increase FOS production [19]. The establishment of this genetic manipulation demonstrated the feasibility of improvement of *A. niger* ATCC 20611 through genetic engineering, which may contribute to reducing the by product generation and simplifying the downstream process.

In this study, the glucose oxidase and peroxidase were heterologously expressed in *A. niger* ATCC 20611 under control of the promoter of the FopA-encoding gene (P*fopA*) and used to accelerate the synthesis of FOS. Meanwhile, to achieve co-localization of the glucose oxidase/peroxidase with FopA in the mycelia, the C-terminal domain of FopA (FopAC) was used to fuse with the glucose oxidase/peroxidase. Then the FOS production was optimized as an integrated process using whole-cells of both the glucose oxidase-expressing strains and the peroxidase-expressing strains, which led to the final FOS product with high concentrations.

## Results

### Construction of the *A*. *niger *strains expressing glucose oxidase under the control of the promoter P*fopA*

**Fig. 1:**
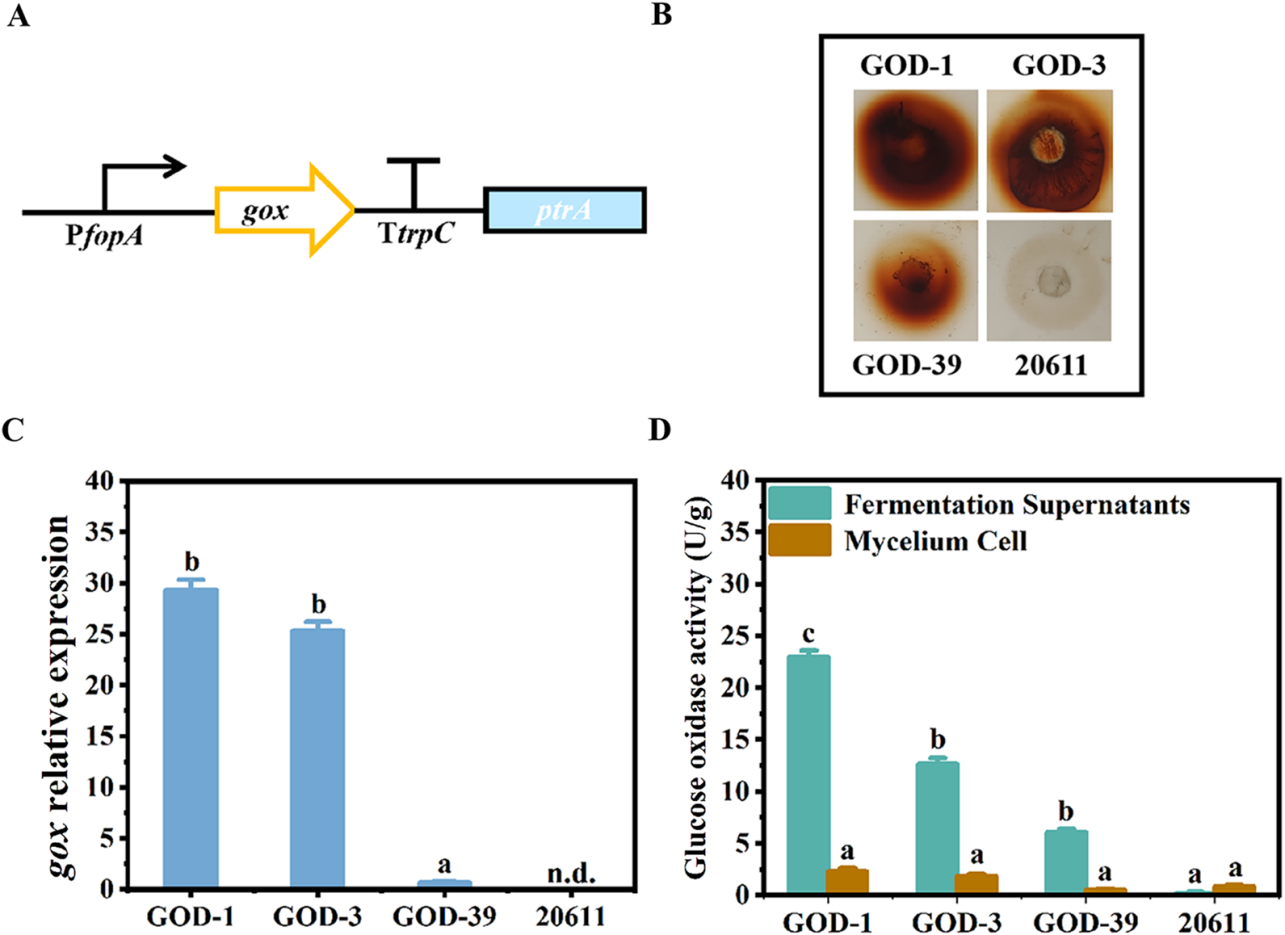
Construction of the glucose oxidase expressing strains of *A. niger*. **A** Schematic diagram of the *gox* expression cassette containing the promoter of the *fopA* gene (P*fopA*), the *gox* gene, the *trpC* terminator (T*trpC*) and the selection marker *ptrA*. The expression cassette was constructed as described in “Materials and methods” section. **B** Assay of glucose oxidase on the GOD-POD bienzymatic detection plate. The same concentrations of spores of the glucose oxidase-expressing transformants GOD-1, GOD-3 and GOD-39 were spotted on the CD plate. Then the amount of glucose released was detected by the GOD-POD bienzymatic system. Glucose oxidase activity was tested by a dark brown halo surrounding the colonies. *A. niger* ATCC 20611 was used as the control. **C** Relative expression levels of *gox* in the glucose oxidase expressing transformants. *Actin* was used as a reference gene and the 2-^ΔΔCt^ method was used for calculating relative expression levels. **D** Determination of the glucose oxidase activities in different cell fractions of transformants. Error bars represent the standard deviation of three independent experiments. “a/b/c” above the bars indicate significant differences at P < 0.01. n.d., not detected. 20611, *A. niger* ATCC20611

Commercially, FOS is generally synthesized from sucrose by β-fructofuranosidase with transfructosylation activity at high sucrose concentrations [6]. However, the FOS content in the β-fructofuranosidase-converted products only reaches up to 55% because of the by-product glucose inhibiting the activity of the enzyme during the reaction [15]. In order to eliminate the by-product glucose, the companies have explored the ways to improve the transfructosylation efficiency. Research shows that the addition of glucose oxidase in the process of FOS synthesis can improve the purity. Until now, the glucose oxidase production using different expression systems has been reported, such as *Escherichia coli* and *Pichia pastoris* [20–22]. However, if the recombinant glucose oxidases are used for FOS synthesis, these enzymes need to be purified and added into the reaction, increasing the cost of FOS production. If glucose oxidase could be heterologously expressed in *A. niger* ATCC 20611, the engineered fungus would co-express β-fructofuranosidase and glucose oxidase, which will synthesize FOS directly without addition of the purified recombinant glucose oxidase. Here, to test the probability of heterologous expression of glucose oxidase in *A. niger* ATCC 20611, the glucose oxidase-encoding gene *gox* from *A. niger* ATCC 1015 was fused with the promoter of the FopA-encoding gene (P*fopA*) and the *trpC* terminator to construct the *gox* expression cassette, which contained the *ptrA* gene as the selectable marker (Fig. 1A). Then, the *gox* expression cassette was transformed into *A. niger* ATCC 20611 and the transformants, which were obtained after screening with the pyrithiamine pressure, were further transferred to the CD plates to detect the glucose oxidase activity. As shown in Fig. 1B, the transformants GOD-1, GOD-3 and GOD-39 showed the remarkable brown halos around the colonies comparing with the parental strain. Subsequently, the genomes of these transformants were analyzed by PCR with the primers gox-F/TtrpC-R. It was found that 2.6 kb of the *gox-trpC* gene could be amplified from the transformants while it was not amplified from the parental strain *A. niger* ATCC 20611, indicating that the *gox* expression cassette was integrated into the genome (Additional file 1: Fig. S1A). Furthermore, the enzyme production of transformant were detected during fermentation. RT-qPCR analysis showed that *gox* was transcribed in the three transformants and the highest transcription level of *gox* was found in the transformant GOD-1 (Fig. 1C). Meanwhile, the activity of glucose oxidase was determined (Fig. 1D). It was found that the transformants exhibited glucose oxidase activity, and the activity mainly existed in the fermentation supernatants while less in mycelia. And the highest glucose oxidase activity reached 22.6 U/g in GOD-1, while no glucose oxidase activity could be detected in the parental strain *A. niger* ATCC 20611. These results demonstrated that glucose oxidase could be successfully expressed in *A. niger* ATCC 20611 under the control of the promoter P*fopA*.

### Construction of the *A. niger* strains expressing glucose oxidase in mycelia

**Fig. 2:**
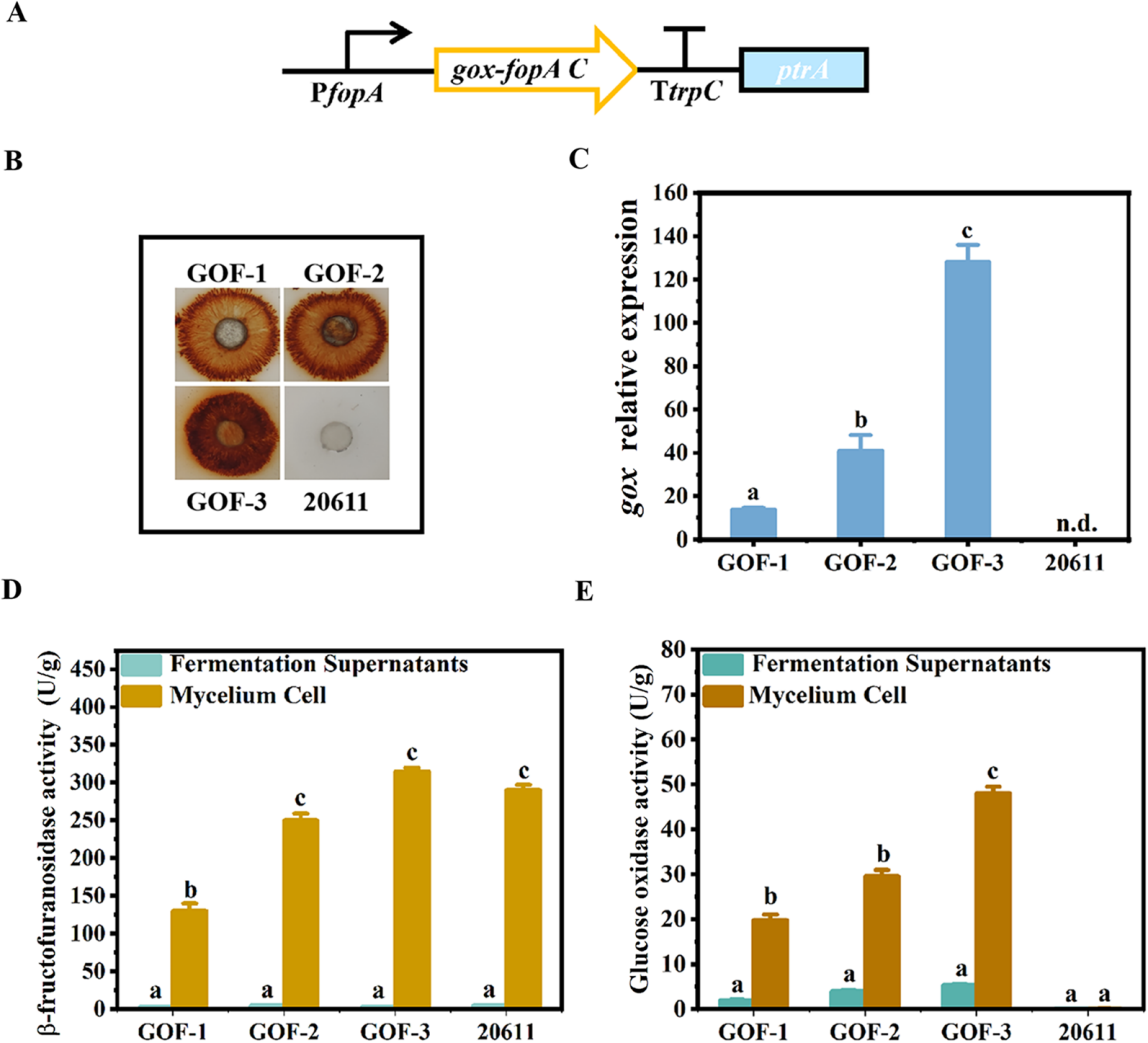
Construction of the glucose oxidase-FopAC expressing strains of *A. niger*. **A** Schematic diagram of the *gox-fopAC* expression cassette containing the promoter of the *fopA* gene (P*fopA*), the *gox* gene, the encoding sequence for the C-terminal domain of the *fopA* (*fopAC*), the *trpC* terminator (T*trpC*), and the selection marker *ptrA*. The expression cassette was constructed as described in “Materials and methods” section. **B** Assay of glucose oxidase activity on the GOD-POD bienzymatic detection plate. The same concentrations of spores of the glucose oxidase-expressing transformants GOF-1, GOF-2 and GOF-3 were spotted on the CD plate. Then the amount of glucose released was detected by the GOD-POD bienzymatic system. Glucose oxidase activity was tested by a dark brown halo surrounding the colonies. *A.niger* ATCC 20611 was used as the control. **C** Relative expression levels of *gox* in the glucose oxidase-FopAC expressing transformants. *Actin* was used as a reference gene and the 2-^ΔΔCt^ method was used for calculating relative expression levels. **D** Determination of fructofructofuranosidase activities of the glucose oxidase-FopAC transformants in different cell fractions. **E** Determination of the glucose oxidase activities in different cell fractions of transformants. Error bars represent the standard deviation of three independent experiments. “a/b/c” above the bars indicate significant differences at P < 0.01. n.d., not detected

It was reported that co-immobilized glucose oxidase and β-fructofuranosidase by sol–gel encapsulation and a relatively high purity of FOS was obtained [23]. Wang et al. used the yeast surface display system to express glucose oxidase on the *Saccharomyces cerevisiae* cells, which created a novel whole-cell biocatalyst [24]. Besides *S. cerevisiae*, four GPI anchored proteins were attempted as cell wall anchoring motifs for the expression of glucose oxidase on other yeast cell surface [25]. Therefore, if glucose oxidase and FopA could be co-expressed, it would facilitate the production of high-purity FOS. FOS could be produced by using the *A. niger* mycelia, because the C-terminal domain of FopA (FopAC) possesses similar function to the cell wall-anchoring domain of the sedimentase in the yeast *S. cerevisiae* [26]. Here, to achieve co-localization of the glucose oxidase with FopA in the mycelia, the FopAC was used to fuse with glucose oxidase to produce the fusion protein glucose oxidase-FopAC. Specifically, the glucose oxidase-FopAC encoding DNA sequence was ligated with the *fopA* promoter and the *trpC* terminator to form the *gox-fopAC* expression cassette, which contained the *ptrA* gene as the selectable marker (Fig. 2A). Subsequently, the expression cassette was transformed into *A. niger* ATCC 20611. The putative transformants were obtained with the screening medium under the pyrithiamine pressure and then transferred to the CD plates to check their glucose oxidase activity. Three transformants GOF-1, GOF-2 and GOF-3 with the largest brown halos were selected (Fig. 2B). Then, the genomes of the transformants were analyzed by PCR with the primers gox-F/FopA-R. The 2.2 kb of the *gox-fopAC* gene could be amplified in the transformants and not in the parental strain, confirming its integration into the genome (Additional file 1: Fig. S1B). RT-qPCR analysis indicated that *gox* was transcribed in the three transformants and the highest transcription level of *gox* was measured in GOF-3 (Fig. 2C). Furthermore, the activity of glucose oxidase and FopA were detected (Fig. 2D, E). It was found that all the three transformants showed glucose oxidase activity, which mainly existed in the mycelia. And the highest glucose oxidase activity reached 49.3 U/g in GOF-3, while no activity was detected in the parental strain. The mycelia-anchored glucose oxidase activity of GOF-3 was approximately 84% of the total enzyme activity (Fig. 2E). Thus, glucose oxidase and fructofuranosidase were successfully co-localized in the mycelial cell. In the transformant GOF-3, both glucose oxidase and FopA displayed mycelial activity, which could catalyze the synthesis of FOS. This novel anchorage position control strategy will enable the efficient utilization of the mycelia of *A. niger* for whole-cell biocatalyst. In addition, the activity of FopA produced by GOF-3 was comparable to that of *A. niger* ATCC 20611, indicating that expression of glucose oxidase didn’t affect the production of FopA. Thus, the engineering strain GOF-3 was selected for the following analysis.

Synthesis of FOS by *A. niger* GOF-3 was carried out, meanwhile the parental strain *A. niger* ATCC 20611 was used for FOS synthesis with/without the commercial glucose oxidase as the control. HPLC analysis was conducted to determine the contents of sugar composition of the reaction mixture and the results of FOS formation were summarized in Table 1. Notably, when the mycelia of GOF-3 was added to the synthesis reaction, the glucose concentration was decreased from 34.16% to 27.72% during the reaction period of 10 h, and the FOS content reached up to 60.63%. While the mycelia of the parental strain ATCC 20611 was added to the synthesis reaction, the glucose formation increased gradually as the reaction progressed and reached 34.16% at 10 h with the final FOS products of 52.07%. Simultaneously, *A. niger* ATCC 20611 was used to synthesize product with the commercial glucose oxidase. The glucose formation was reduced to 27.97% at 10 h of reaction, while the FOS content reached 59.31%, which was lower than the yield of GOF-3 (60.63%) (Table 1). The increase in the content of FOS synthesized by GOF-3 was probably due to the lack of inhibition of FopA by glucose, which was removed by glucose oxidase. Taken together, *A. niger* GOF-3 not only successfully expressed glucose oxidase, but also facilitated the FOS synthesis. Thus, *A. niger* GOF-3 displayed an enhanced capacity for FOS production. However, there still existed substantial glucose (30%) in its reaction solution, which needed to be further eliminated for improvement of FOS synthesis.

**Table 1:** HPLC analysis of FOS yield and composition after 10 h reaction

Strain	Glucose (%)	Fructose (%)	Sucrose (%)	GF_2_ (%)	GF_3_ (%)	GF_4_ (%)	FOS (%)
20611	32.16 (± 0.13)^a^	1.30 (± 0.09)	14.47 (± 0.18)	21.32 (± 1.30)	24.66 (± 1.27)	5.09 (± 0.06)	52.07 (± 0.61)
20611 + GOX	27.97 (± 0.21)	1.23 (± 0.06)	11.50 (± 0.15)	25.84 (± 0.50)	26.93 (± 1.28)	6.54 (± 0.07)	59.31 (± 0.27)
GOF-3	27.72 (± 0.25)	1.16 (± 0.13)	10.49 (± 0.61)	27.22 (± 0.31)	26.88 (± 1.01)	6.53 (± 0.02)	60.63 (± 0.36)

All reactions were carried out in a 40% (W/V) sucrose solution at 40 °C, with shaking at 120 rpm, the enzyme dosage was 6 U per g sucrose for the parental strain and equal amounts of the transformant cells

^a^Values in parentheses are standard errors

### Construction of the *A. niger* strains expressing peroxidase under the control of the promoter P*fopA*

**Fig. 3:**
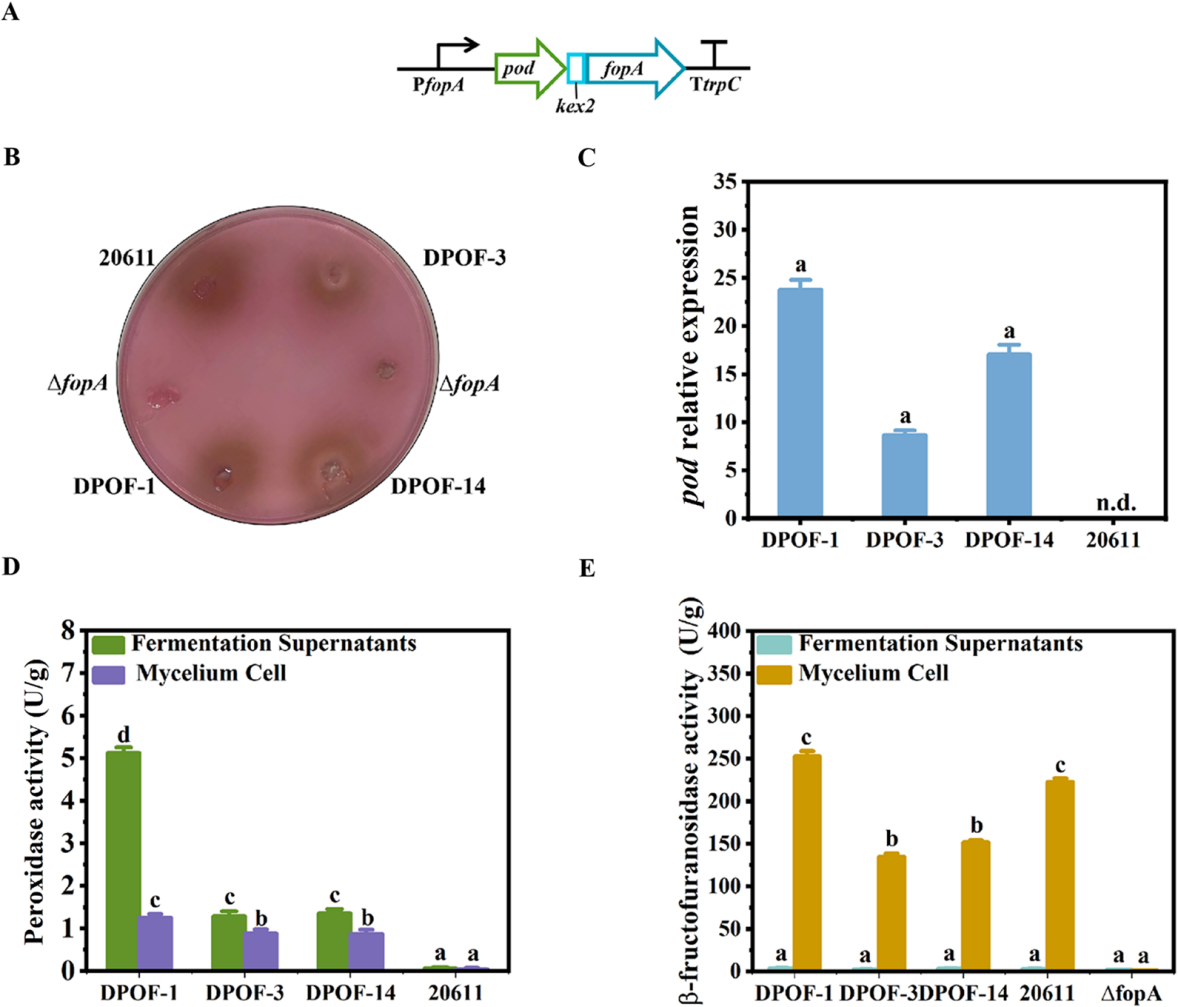
Construction of the peroxidase-FopA expressing strains of *A. niger*. **A** Schematic diagram of the *pod-fopA* expression cassette containing the promoter of the *fopA* gene (P*fopA*), the horseradish roots *pod* gene, the *fopA* sequence and the *trpC* terminator (T*trpC*). The expression cassette was constructed as described in “Materials and methods” section. **B** Assay of peroxidase and FopA on the GOD-POD bienzymatic detection plate. The same concentrations of spores of the peroxidase-expressing transformants DPOF-1, DPOF-3 and DPOF-14 were spotted on the CD plate. Then the amount of glucose released was detected by the GOD-POD bienzymatic system. Peroxidase activity was tested by a dark brown halo surrounding the colonies. *A.niger* ATCC 20611 and *A. niger ∆fopA* were used as the control. **C** Relative expression of *pod* in the peroxidase-FopA expressing transformants. *Actin* was used as a reference gene and the 2-^ΔΔCt^ method was used for calculating relative expression levels. **D** Determination of the peroxidase activities in different cell fractions of transformants using glucose as the substrate. **E** Determination of fructofructofuranosidase activities of the peroxidase-FopA transformants in different cell fractions by ABTS method. Error bars represent the standard deviation of three independent experiments. “a/b/c” above the bars indicate significant differences at P < 0.01. n.d., not detected

It is known that hydrogen peroxide would be produced during the oxidation of glucose by glucose oxidase, which has an inhibitory effect on the catalytic activity of glucose oxidase [17]. Thus, it can be speculated that addition of peroxidase into the FOS synthesis reaction would eliminate hydrogen peroxide to facilitate the removing of glucose. In addition, peroxidase has been reported to be expressed through recombinant DNA technology in fungi, especially *Pichia pastoris* [27, 28]. Thus, in order to express peroxidase in *A. niger* ATCC 20611 to facilitate the FOS synthesis, a peroxidase-encoding gene (*pod*) from horseradish roots was linked with the full-length FopA-encoding gene (*fopA*, as a fusion reporter gene) by the DNA sequence encoding a recognition site (*kex2*) for a fungal processing protease (Kex2) to allow in vivo splicing of the fused protein peroxidase-FopA. In addition, the promoter P*fopA* and the *trpC* terminator were used to control the expression of the *pod-fopA* gene. And the *pod-fopA* expression cassette was shown in Fig. 3A. Then, the expression cassette was transformed into the *A. niger ∆fopA*, which was a *fopA* knock-out strain of *A. niger* ATCC 20611 (unpublished data). Thus, the transformants could be screened on the CD plates to detect the FopA activity based on the GOD-POD bienzymatic system [29]. Three transformants, DPOF-1, DPOF-3 and DPOF-14, which colonies were surrounded by the clear pink halos, were selected for further analysis (Fig. 3B). Then, the transformants were analyzed by PCR to amplify the *pod* gene with the primers pod-F/pod-R (Additional file 1: Fig. S2A). The 1.2 kb of the *pod* gene could be amplified in the transformants but not in the parental strain, indicating the integration of the expression cassette into the genome of the transformants. RT-qPCR analysis showed that *pod* was transcribed in the three transformants (Fig. 3C). Furthermore, the transformants were fermented to detect the activities of peroxidase and FopA (Fig. 3D, E). It was found that all the three transformants showed peroxidase activity, which mainly existed in the fermentation supernatants. Among them, the transformant DPOF-1 has the highest peroxidase activity (5.1 U/g), while the parental strain did not show any activity. In addition, the activity of FopA in DPOF-1 was similar to that in *A. niger* ATCC 20611, while no activity was found in *A. niger ∆fopA*. These results demonstrated that the fusion expression cassette was integrated into the genome and the peroxidase was successfully expressed in *A. niger* ATCC 20611.

### Construction of the *A. niger* strains expressing peroxidase in mycelia

**Fig. 4:**
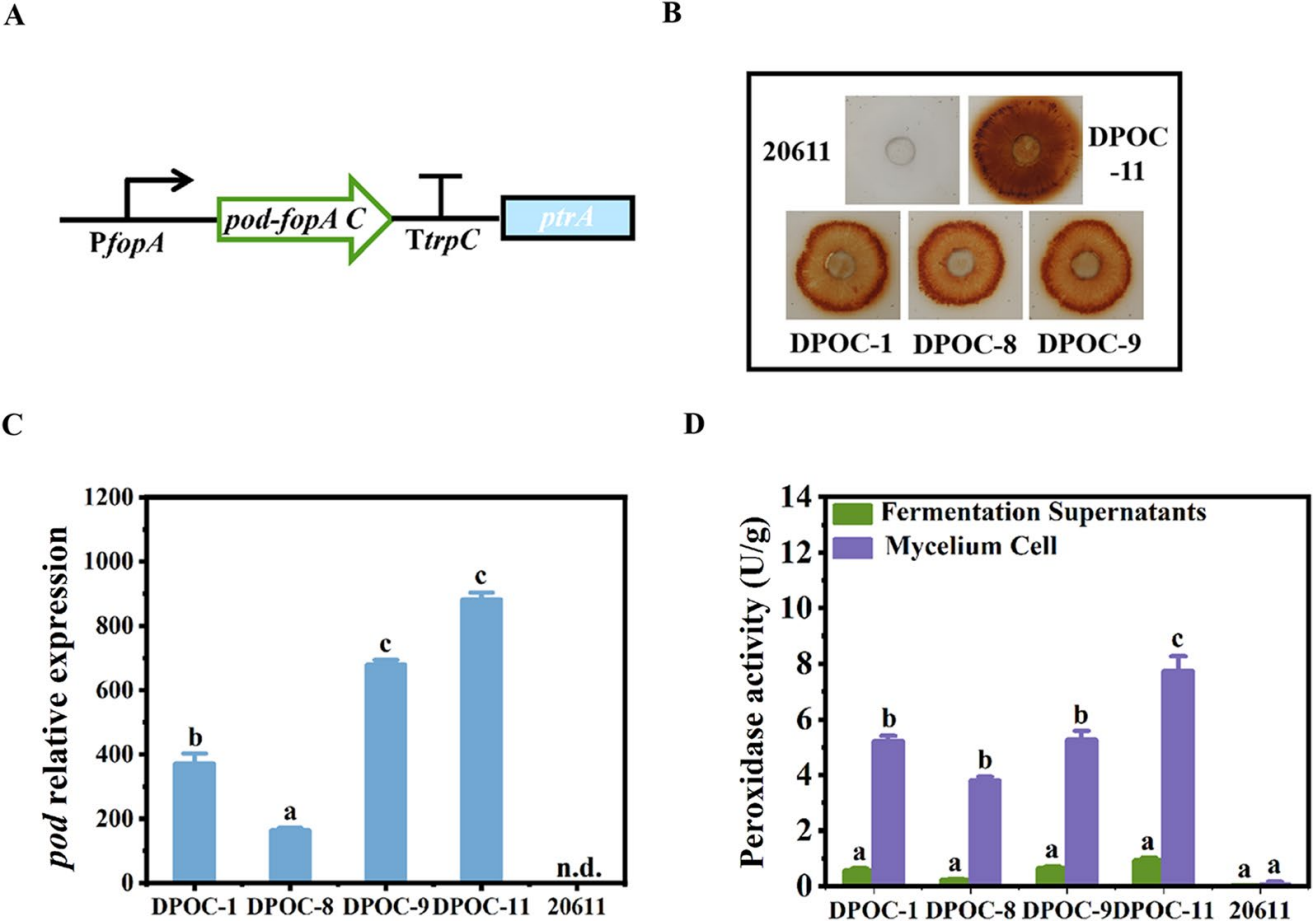
Construction of the peroxidase-FopAC expressing strains of *A. niger.* **A** Schematic diagram of the *pod-fopAC* expression cassette containing the promoter of the *fopA* gene (P*fopA*), the horseradish roots *pod* gene, the encoding sequence of the C-terminal domain of the *fopA* (*fopAC*), the *trpC* terminator (T*trpC*), and the selection marker *ptrA*. The expression cassette was constructed as described in “Materials and methods” section. **B** Assay of peroxidase on the GOD-POD bienzymatic detection plate. The same concentrations of spores of the peroxidase-expressing transformants DPOC-1, DPOC-8, DPOC-9 and DPOC-11 were spotted on the CD plate. Then the amount of glucose released was detected by the GOD-POD bienzymatic system. Peroxidase activity was tested by a dark brown halo surrounding the colonies. *A. niger* ATCC 20611 was used as the control. **C** Relative expression of *pod* in the peroxidase-FopAC expressing transformants. *Actin* was used as a reference gene and the 2-^ΔΔCt^ method was used for calculating relative expression levels. **D** The peroxidase activities in the transformants. Error bars represent the standard deviation of three independent experiments. “a/b/c” above the bars indicate significant differences at P < 0.01. n.d., not detected

Although peroxidase can be expressed in *A. niger* ATCC 20611 when fused with the full-length FopA under the control of the promoter P*fopA*, the peroxidase of the transformants mainly existed in fermentation supernatants (Fig. 3D). Since synthesis of FOS was made by the *A. niger* mycelia, the peroxidase needed to be expressed in mycelia. Thus, peroxidase and the FopA C-terminal domain FopAC were fused to produce the fusion protein POD-FopAC. Specifically, the fusion protein POD-FopAC encoding DNA sequence (*pod-fopAC*) containing the peroxidase-encoding senquence (*pod*) and the *fopAC* sequence was ligated with the P*fopA* promoter and the *trpC* terminator to form the *pod-fopAC* expression cassette, which also contained the *ptrA* gene as the selectable marker (Fig. 4A). Then, the expression cassette was transformed into *A. niger ∆fopA*. Transformants were screened on the CD medium containing pyrithiamine as the selection pressure and the MM medium based on the oxidation of *o*-anisidine for detection of the peroxidase production. The transformants DPOC-1, DPOC-8, DPOC-9 and DPOC-11 showed intense purple halos around the colonies, indicating that these transformants produced peroxidase (Fig. 4B). Then, the transformants were analyzed by PCR to amplify 1.7 kb of the *pod-fopAC* gene with the primers pod-F/ fopA-R. The *pod* gene could be amplified in the transformants but not in the parental strain, indicating the integration of the expression cassette into the genome of the transformants (Additional file 1: Fig. S2B). RT-qPCR analysis showed that *pod* was transcribed in the four transformants and the highest transcription level of *pod* was found in DPOC-11 (Fig. 4C). Furthermore, the activity of peroxidase was detected (Fig. 4D). All the transformants showed the peroxidase activity, which mainly existed in the mycelia. And the highest peroxidase activity reached 7.9 U/g in DPOC-11, while no activity was present in the parental strain. These results demonstrated that peroxidase was successfully expressed in the mycelia of *A. niger*. And the transformant DPOC-11 was selected for the following analysis.

The transformant DPOC-11 was cultivated on the modified CD plate containing FOS, glucose and sucrose to determine the carbon source utilization (Additional file 1: Fig. S3). It was found that DPOC-11 and its parental strain *∆fopA* could grow on the glucose-plate and were almost not able to grow on the FOS/sucrose-plate. These results indicated that DPOC-11 could not use FOS and sucrose as the carbon source. Meanwhile, the wild-type strain ATCC 20611 could use all the three carbon sources. Thus, it was speculated that DPOC-11 could remove glucose and avoid consumption of FOS if it was used in the process of FOS synthesis, which would facilitate the production of high-purity FOS. In the following experiments, the transformant DPOC-11 was used for FOS synthesis.

### Synthesis of FOS by the glucose oxidase expressed in *A. niger* GOF-3 and the peroxidase expressed in strain* A. niger* DPOC-11

**Fig. 5:**
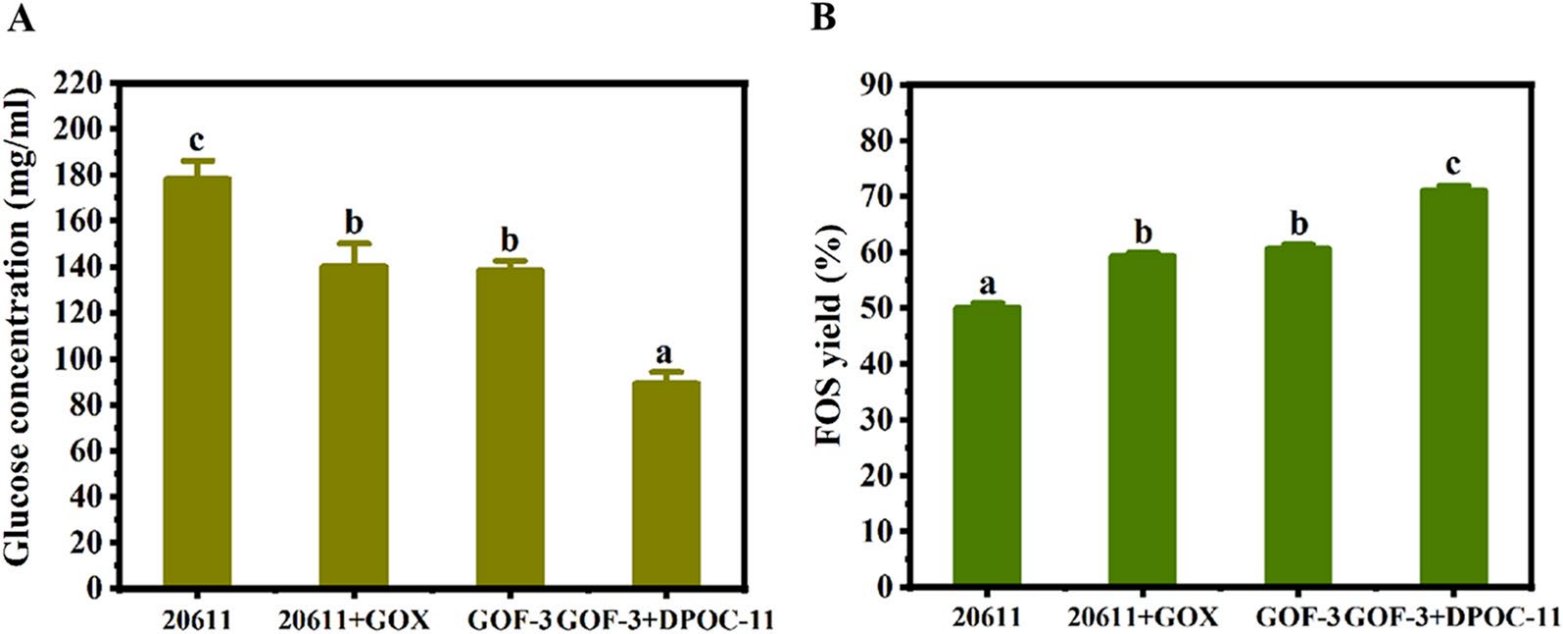
The production of FOS by using the glucose oxidase-expressing strain *A. niger* GOF-3 and the peroxidase-expressing *A. niger* DPOC-11. **A** The glucose content in the product formed by *A. niger* GOF-3 and DPOC-11 during the transglycosylation reaction using 40% (w/w) sucrose as substrate. *A. niger* ATCC 20611, *A. niger* ATCC 20611 plus glucose oxidase (20611 + GOX) and *A. niger* GOF-3 were used as the controls. **B** The yield of FOS produced by *A. niger* ATCC 20611, *A. niger* ATCC 20611 plus glucose oxidase (20611 + GOX), *A. niger* GOF-3 and *A. niger* GOF-3 plus DPOC-11 (GOF-3 + DPOC-11) during the transglycosylation reaction using 40% (w/w) sucrose as substrate. The products were detected by HPLC. Error bars represent the standard deviation of three independent experiments. “a/b/c” above the bars indicate the significant differences at P < 0.01

Since glucose oxidase was successfully co-expressed with FopA in the mycelia of *A. niger* GOF-3, when the engineering strain GOF-3 was used for the FOS synthesis, hydrogen peroxide would be produced, which has an inhibitory effect on the catalytic activity of glucose oxidase [17]. Furthermore, if the peroxidase-expressing strain DPOC-11 was used in the process of FOS synthesis, the by-product glucose would be eliminate and the high-purity-FOS may be obtained. Firstly, the mycelia of GOF-3 was used for synthesis reaction. Then the mycelial cells of DPOC-11 were added to reaction mixture after 3 h of the reaction (Additional file 1: Fig. S4). HPLC analysis was conducted to determine the contents of sugar composition of the reaction mixture (Fig. 5). Notably, when the transformants GOF-3 and DPOC-11 were used to jointly synthesize FOS, the glucose content in the product after 10 h decreased to 89.33 mg/ml, which was 49.91% lower than that of the parental strain, and the FOS content reached 71.00%. When the mycelia of GOF-3 was added to the synthesis reaction, the content of glucose in the product was 138.62 mg/ml after 10 h, which was 22.27% lower than that of *A. niger* ATCC 20611, and the content of FOS was 60.63%. When the mycelia of *A. niger* ATCC 20611 was used to synthesize FOS with the commercial glucose oxidase. After reacting for 10 h, the glucose content decreased to 140.16 mg/ml, and the FOS content increased to 59.31% at this time. When only *A.niger* ATCC 20611 participated in the reaction for 10 h, the glucose concentration reached 178.33 mg/ml and the FOS content was 50.07%. Taken together, these results demonstrated that the glucose oxidase-expressing strain GOF-3 and the peroxidase-expressing strain DPOC-11 could be used to produce the enhanced concentration of FOS.

## Discussion

For more than decades, the fructooligosaccharides (FOS) have drawn special attention due to their functional properties of selectively invigorating the growth of beneficial microorganisms in the colon [30]. *A. niger* ATCC 20611 is the common industrial fungus used for FOS production due to the FopA possessing a high transfructosylation activity, but the yield of FOS was generally no more than 55% (w/w) conversion [19]. Therefore, commercial production of high-purity FOS has been an important direction for the development of FOS industry [4]. However, the production of high-purity FOS at present is an expensive procedure including chromatographic methods or microfiltration purification [13]. This study aimed to genetically engineer the industrial fungus *A. niger* ATCC 20611 to produce high-purity FOS by expressing glucose oxidase and peroxidase using the promoter P*fopA* to consume the by-product glucose, thus avoiding the costly physical separation process.

Gene expression in filamentous fungi is regulated primarily at the level of transcriptional initiation [31]. Using strong promoters to drive gene expression is an efficient method to increase heterologous protein production. So far, the glyceraldehyde-3-phosphate dehydrogenase promoter P*gpdA* of *A. nidulans* is the most frequently used for gene expression in *Aspergillus* [32]. However, when this promoter was used for overexpression of FopA, the production of FopA was only increased by less than onefold [20]. More recently, it was found that the promoter of the FopA gene (P*fopA*) was an inducible promoter by sucrose, which is 10 times stronger than P*gpdA* [33]. Here, the promoter P*fopA* was used to express the *gox* gene in *A. niger* ATCC 20611. And the transformant GOF-3 showed the glucose oxidase activity reaching to 49.3 U/g at 48 h (Fig. 2D). In addition, the FopA in the transformed strain GOF-3 is consistent with that of the parental strain (Fig. 2E), indicating that the expression of the *gox* gene could not influence the expression of the *fopA* gene. These results demonstrated that P*fopA* is appropriate for efficient expression of heterolgous proteins in *A. niger* ATCC 20611.

As reported, the display of enzymes on the microbial cell surface prove to be a novel protein immobilization method with whole cells as carriers [34]. Compared with the conventional immobilization methods, this strategy has advantages such as better biocatalyst stability, reduction of mass transfer limitations, or the possibility of reuse in several repeated runs [35]. Proteins could be directly immobilized on the microbial cell surface by fusing an anchoring motif to the N- or C-terminus of the protein [36]. Wang et al. used the yeast surface display system to express glucose oxidase on the *S. cerevisiae* cells, which created a novel whole-cell biocatalyst [25]. Besides *S. cerevisiae*, four GPI anchored proteins were attempted as cell wall anchoring motifs for the expression of glucose oxidase on other yeast cell surface [26]. In this study, the C-terminal domain of the FopA (FopAC) was attempted to immobilize glucose oxidase/peroxidase on the mycelial cell wall of *A. niger* ATCC 20611. As shown in Fig. 2E, the mycelia-anchored glucose oxidase activity of *A. niger* GOF-3 was approximately 84% of the total enzyme activity. Thus, glucose oxidase was successfully co-located with the native fructofuranosidase in the mycelial cell of *A. niger* GOF-3. This novel anchorage position control strategy will enable the efficient utilization of the mycelia of *A. niger* for whole-cell biocatalyst. In addition, the mycelia has the potential to be recycled to achieve continuous synthesis of FOS, which would further facilitate the industrial production of FOS. During the production of FOS, large amounts of by product glucose is generated and inhibits the activity of FopA, resulting in the relatively low concentration of FOS (no more than 55%). The strategies for removal of glucose were by means of various purification technologies, such as membrane technologies, chromatographys, microbial fermentation and enzymatic treatment [37–39]. Membrane technologies and chromatographys require multiple repeated operations and Instruments and equipment, which greatly increased the cost of production [39]. The glucose can be converted into precipitates for removal by the enzymatic treatment [40]. Valdivieso-Ugarte et al. added the extracellular glucose oxidase to the FOS synthesis reaction, and the final syrup reaching up to 90% of FOS was obtained [15]. However, these processes increased process complexity while pushing up the cost. This study expressed glucose oxidase to oxidate most of glucose present in the FOS synthesis mixture, thus increasing the concentration of FOS (60.63%). Moreover, microbial treatment is a considerable way to remove glucose. Fan et al*.* used *Bacillus coagulans* to decrease the abundance of glucose during the production of FOS [41]. There are reports where the species such as *Saccharomyces cerevisiae*, *Kluyveromyces lactis* and *Pichia pastoris* were used to convert monosaccharides and disaccharides into ethanol and carbon dioxide and/or glycerol, but ethanol and glycerol need extra processing to be removed [38, 42, 43]. Here, we found that the peroxidase-expressing strain *A. niger* DPOC-11 does not utilize FOS and is therefore ideal for the purification of glucose with no unwanted by product produced. Specifically, the peroxidase expressed in *A. niger* DPOC-11 would prolong the oxidation action of glucose by glucose oxidase with elimination of hydrogen peroxide, thus facilitating the production of high-purity FOS (71.00%). Although the stability of the enzymes produced by the engineered strains is still need to be verified before the large-scale production, the mycelial FopA was reported to be capable of being recycled for the FOS synthesis. It was found that the enzyme still had 62.3% of initial activity after being reused for six consecutive cycles in the parental strain ATCC 20611 [18]. Taken together, *A. niger* GOF-3 and *A. niger* DPOC-11 displayed a preferable effectiveness for FOS production. And this type of purification process led to produce the final FOS products with increased concentrations, which would contribute to decreasing the capital and operating costs.

## Conclusion

In this study, the glucose oxidase and peroxidase were heterologously expressed in an industrially important β-fructofuranosidase-producing fungus *A. niger* ATCC 20611, which was the main commercial FOS producer. Particularly, the heterologous enzymes were successfully detected in the mycelia because of the fusion with the FopAC domain of the native β-fructofuranosidase. The multiple-enzyme producing mycelia of these strains could be directly applied to convert the sucrose without additional purification procedures, leading to a final product with high concentration of FOS (71.00%). This is expected to be a cost-effective and efficient FOS production technology with whole-cell catalysis. This study not only facilitated the high-purity-FOS synthesis, but also demonstrated the potential of *A. niger* ATCC 20611 as an enzyme-producing cell factory.

## Methods

### Strains and media

*Aspergillus niger* ATCC 20611, an important industrial FOS-producing strain, was used as the parental strain for the expression of glucose oxidase and peroxidase. To collect spores, the strains were cultured on potato dextrose agar medium (PDA, 20 g d-glucose/l, 200 g potato/l, and 20 g agar/l). Selection of transformants was carried out on minimal medium (MM) with 2 μg/ml pyrithiamine. The fermentation medium (FM) contained 15 g yeast extract/l, 0.5 g MgSO_4_. 7H_2_O/l, 0.5 g KCl/l, 5 g K_2_HPO_4_/l and 2 g NaNO_3_/l, 50 g sucrose/l at pH 5.0. Czapek Dox (CD) agar medium consisted of NaNO_3_ 3 g/l, K_2_ HPO_4_ 1 g/l, MgSO_4_·7H_2_O 0.5 g/l, KCl 0.5 g/l, FeSO_4_ 0.01 g/l, CuSO_4_ 0.01 g/l, sucrose 30 g/l and agar 20 g/l.

### Construction of the expression cassettes

Two *gox* and two *pod* expression cassettes were constructed using a PCR cloning approach, and the cloned PCR products were checked by sequencing. The P*fopA*-*gox*-T*trpC*-*ptrA* expression cassette for expression of *gox* in *A. niger* was constructed as follows. The *fopA* promoter (P*fopA*) with signal peptide-encoding DNA sequence was amplified with the primer pairs fopA-UF/fopA-60R using the *A. niger* ATCC 20611 genomic DNA as the template. The *gox* gene was amplified from the genome of *A. niger* ATCC 1015 using primer pairs gox-F/gox-1815R. The DNA fragment containing the efficient transcription terminator *trpC* (T*trpC*) was amplified with the primer pairs TtrpC-F/TtrpC-R using the plasmid pAN7-1 as the template. Then, the *fop*A promoter, the *gox* gene, the *trpC* terminator and the *ptrA* sequence were fused into the expression cassette by Double-joint PCR and amplified with primers fopA-UF/TtrpC-R.

The P*fopA*-*gox*-*fopAC*-T*trpC*-*ptrA* expression cassette for expression in *A. niger* was constructed as follows. The *fopAC* sequence was amplified with the primer pairs fopA-1438F/fopA-R using the *A. niger* ATCC 20611 genomic DNA as the template. Then, the *fopA* promoter, the *gox* gene, the *fopAC* sequence, the *trpC* terminator and the *ptrA* sequence were fused into the expression cassette by Double-joint PCR and amplified with primers fopA-UF/ptrA-R.

The P*fopA*-*pod*-*fopA*-T*trpC* expression cassette for expression in *A. niger* was constructed as follows. The pod sequence was amplified with the primer pairs pod-F/pod-R using the plasmid pU-POD as the template. The *fopA* was amplified from *A. niger* ATCC 20611 genome using primer pairs fopA-F/fopA-R. Finally, Double-joint PCR technology was used to fuse the expression cassette, containing the *fopA* promoter, the *pod* gene, the *fopA* sequence and the *trpC* terminator. A DNA sequence encoding a recognition site for a fungal processing protease was placed between the *pod* and the *fopA* sequences to allow in vivo splicing of the fused proteins [44].

The P*fopA*-*pod*-*fopA*C-T*trpC*-*ptrA* expression cassette for expression in *A. niger* was constructed as follows. The *fopA* promoter, the *pod* gene, the *fopAC* sequence, the *trpC* terminator and the *ptrA* sequence were fused into the expression cassette by Double-joint PCR and amplified with primers fopA-UF/ptrA-R. All PCR primers were designed using Primer Premier 5.0 software. Additional file 1: Table S1 summarizes the Primers given above.

### Transformation of *A. niger* ATCC 20611

Protoplasts were prepared as the described method with modifications [45]. The mycelia were added into 20 ml of Solution I (1.2 M sorbitol-0.1 M KH_2_PO_4_, pH 5.6), in which the cell wall-digesting enzymes were dissolved at appropriate concentrations, including the *Trichoderma harzianum* lysing enzyme (Sigma Corp., St Louis, MO), snailase (Dingguo Corp., Beijing, China) and lysozyme (Genview, Houston, TX). Incubation was performed at 28 °C with gentle shaking for 2 h. Then the mixtures were filtered aseptically through 4 layers of glass wool and centrifuged at 2500 rpm to collect the protoplasts. Then the expression cassettes were transformed into *A. niger* ATCC 20611 according to the previously described method [20].

### Activity assay of glucose oxidase

Glucose oxidase activity was determined according to the method described by Valdivieso-Ugarte. The mycelial cells were collected for glucose oxidase assay after incubation in 500-ml shaken flask containing 200 ml of FM at 30 °C for 48 h. In brief, 20 µl of samples were incubated in 180 µl of reaction mixture, which contained 20 mg/ml glucose, 0.08 mg/ml *o*-dianisidine, 0.6 U/ml horseradish peroxidase in 100 mM citric acid-disodium hydrogen phosphate buffer pH 5.0 (100 mM McIlvain buffer). The mixture was incubated at 37 °C for 15 min, then absorbance was measured at 415 nm against a standard curve with hydrogen peroxide of different concentrations reacted in the same way. One unit of glucose oxidase activity (U) was defined as the amount of enzyme required to oxidize 1 μmol of glucose per min. To check glucose oxidase activity on solid plates, colonies grown on MM plates were picked on CD plates, incubated 24 h at 30 °C, and finally overlaid with 8 ml of reaction mixture (10 mg/ml glucose, 0.6 U/ml peroxidase, 0.08 mg/ml *o*-dianisidine) in 1% agarose prepared with pH 5.0 citric acid buffer. Plates were incubated 2–4 h at 30 °C. Enzyme activity was detected by a dark brown halo surrounding the colonies.

### Activity assay of peroxidases

Peroxidases activity was measured according to the method described by Glenn [46]. In short, the mycelia cells were taken into reaction mixture for monitoring the oxidation of diammonium 2, 2′-azinobis (3-ethylbenzthiazoline-6-sulfonate) (ABTS) in the presence of 1.5 mM H_2_O_2_ at 415 nm. Similarly, peroxidase activity was screened on solid plates. Colonies grown on MM plates were picked on CD plates. The plates were incubated at 30 °C for 24 h and then flooded with a solution of 0.08 mg/ml *o*-dianisidine, 10 mg/ml glucose and 10 U/ml glucose oxidase. The peroxidase-expressing transformants would produce a dark brown halo upon incubation at 30 °C.

### Activity assay of β-fructofuranosidase

The fresh conidia (1 × 10^6^/ml) of the transformants were inoculated into 200 ml of fermentation medium, containing 50 g sucrose/l, 15 g yeast extract/l, 0.5 g KCl/l, 0.5 g MgSO_4_. 7H_2_O/l, 5 g K_2_HPO_4_/l and 2 g NaNO_3_/l (pH 5.0). After incubation for 48 h at 30 °C under shaking with a speed of 200 r/min. The mycelial cells were obtained through centrifugation for the β-fructofuranosidase activity assay. The activity assay was carried out according to the 3,5-dinitrosalicylic acid (DNS) method as described previously [20].

### Enzyme activity in different cell fractions of the transformants

The mycelia were added into 20 ml of Solution I (1.2 M sorbitol-0.1 M KH_2_PO_4_, pH 5.6), in which the cell wall-digesting enzymes were dissolved at appropriate concentrations. Incubation was performed at 28 °C with gentle shaking for 2 h. Then the mixtures were isolated by centrifugation at 2500 rpm for 10 min, in which supernatant was collected for determination of the enzyme activity of the cell-wall associated fractions, and protoplasts were used for the determination of intracellular enzyme activity. Glucose oxidase, peroxidase activity and fructofuranosidase activity in different cell fractions of the transformants were determined by the method given above. Each strain performed three biological replicates and showed the data as the mean and SD of three replicate cultures.

### The transformants for the production of FOS

The reaction mixture for FOS production consisted of the collected mycelial cells of *A. niger*, 40% sucrose, 0.1% CaCO3 in 50 mM citrate phosphate buffer (pH 5.5), and was incubated on a shaker at 40 °C. The mixture was taken at appropriate times and the supernatant was collected by filtration. The addition of CaCO_3_ neutralized the gluconic acid produced by the glucose oxidase, which helped maintain the pH of the reaction mixture and facilitated synthesis of FOS. The concentration of sample was analyzed by an high-performance liquid chromatography system (HPLC, LC-6A, Shimadzu, Japan) equipped with a refractive index detector (2414, Waters, USA) and a Agilent Zorbax NH_2_ column (5 μm, 4.6 mm × 250 mm) (Agilent Technologies, Santa Clara, CA). The reaction was performed with multiple parallel samples.

### HPLC analysis

The concentration of the reaction products was analyzed by a high-performance liquid chromatography system (HPLC, LC-6A, Shimadzu, Japan), which was equipped with a refractive index detector (2414, Waters, USA) and a Agilent Zorbax NH_2_ column (5 μm, 4.6 mm × 250 mm) (Agilent Technologies, Santa Clara, CA). The column was eluted with 70% (v/v) acetonitrile at a flow rate of 1 ml/min at 30 °C. The determination and quantification of the reaction products were carried out against the standard curve, which was established based on standards of glucose, fructose, sucrose, 1-kestose, nystose and 1^F^-fructofuranosylnystose (Sigma Corp, St Louis, MO). Before loading, samples should be appropriately diluted and filtered (0.22 lm membrane).

### Supplementary Information

**Additional file 1: Table S1.** Primers used in this study; **Fig. S1.** PCR analysis of the glucose oxidase-expressing transformants of *A. niger*; **Fig. S2.** PCR analysis of the peroxidase-expressing transformants of *A. niger*; **Fig. S3.** The carbon source utilization of the peroxidase-FopAC expressing strain *A. niger* DPOC-11; **Fig. S4.** Schematic diagram of the process of FOS synthesis.

## Data Availability

The datasets used and analyzed during the current study are available from the corresponding author upon reasonable request.

## Abbreviations

FOS: Fructooligosaccharides
FopA: β-Fructofuranosidase
P*fopA*: The promoter of the FopA gene
P*gpdA*: The promoter of glyceraldehyde-3-phosphate dehydrogenase gene
