# Supplementary material for: Effective synthesis of high-content fructooligosaccharides in engineered Aspergillus niger
Source: Microb Cell Fact. 2024 Mar 9;23:76. doi: 10.1186/s12934-024-02353-w (PMC10924377; doi:10.1186/s12934-024-02353-w)
Supplement: Supplementary file 1 — Additional file 1: Table S1. Primers used in this study; Fig. S1. PCR analysis of the glucose oxidase-expressing transformants of A. niger; Fig. S2. PCR analysis of the peroxidase-expressing transformants of A. niger; Fig. S3. The carbon source utilization of the peroxidase-FopAC expressing strain A. niger DPOC-11; Fig. S4. Schematic diagram of the process of FOS synthesis. [file 12934_2024_2353_MOESM1_ESM.docx]

**Additional file**

**Table S1** Primers used in this study.

| Primers | Sequences (5’-3’) | Genes-regions |
| --- | --- | --- |
| fopA-UF | ATTGTGTCATTATAGCTCTTTCT | Promoter of *fopA* |
| fopA-60R | TGAGGCTTCTGCTGCTGC | Promoter of *fopA* |
| gox-F | ATGCAGACTCTCCTTGTGAGCT | *gox* |
| gox-1815R | CTGCATGGAAGCATAATCTTCC | *gox* |
| pod-F | CAGCTCACCCCTACGTTT | *pod* |
| pod-R | CATGCTCGAGACAAAGT | *pod* |
| fopA-F | ATGAAGCTCACCACTACCACCC | *fopA* |
| fopA-R | TCAATTTCTCTCCGGCCAG | *fopA* |
| ptrA-F | CCCCAGGCTTTACACTTTAT | *ptrA* |
| ptrA-R | GCGGCTCATCGTCACCCCAT | *ptrA* |
| trpC-F | ACTTAACGTTACTGAAATCATCAAA | Terminator of *trpC* |
| trpC-R | GAGTGGAGATGTGGAGTGGG | Terminator of *trpC* |
| fopA-1438F | TCGGTGACCGCGGAGGAG | *fopAC* |

###
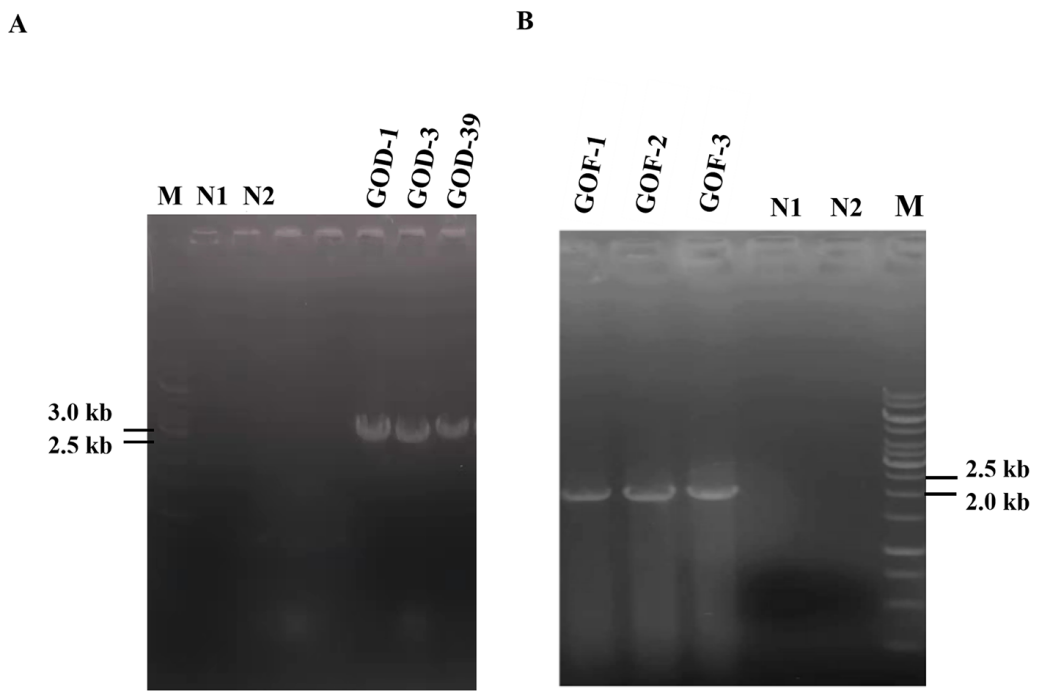


### Fig. S1. PCR analysis of the glucose oxidase-expressing transformants of *A. niger*. M, marker; N1, H_2_O; N2, *A.* *niger* ATCC 20611. GOD-1, GOD-3 and GOD-39 were the glucose oxidase-expressing transformants. GOF-1, GOF-2 and GOF-3 were the glucose oxidase-FopAC-expressing transformants.


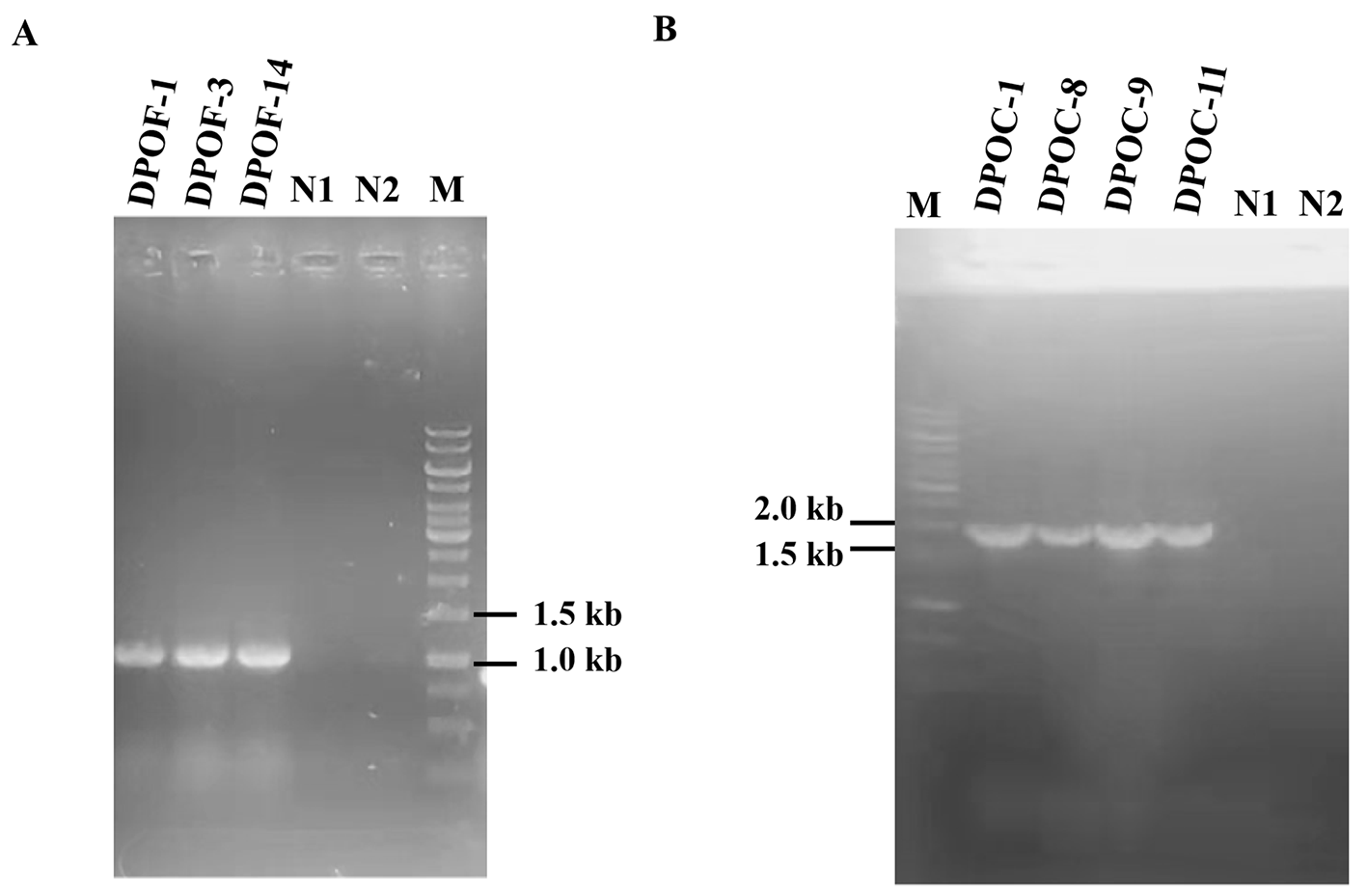


### Fig. S2. PCR analysis of the peroxidase-expressing transformants of *A. niger*. M, marker; N1, H_2_O; N2, *A.* *niger* ATCC 20611. DPOF-1, DPOF-3 and DPOF-14 were the peroxidase-expressing transformants. DPOC-1, DPOC-8, DPOC-9 and DPOC-11 were the peroxidase-FopAC-expressing transformants.


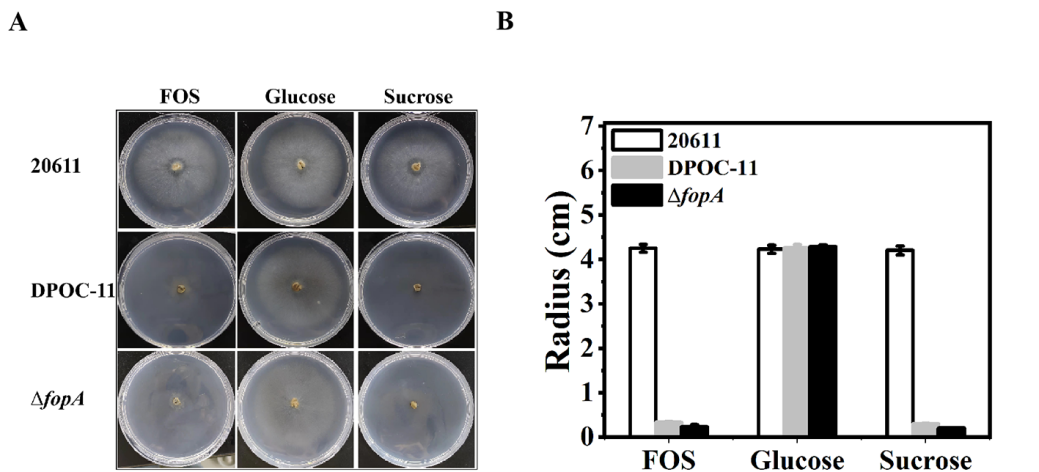


**Fig. S3.** The carbon source utilization of the peroxidase-FopAC expressing strain *A. niger* DPOC-11. A. The carbon source utilization of DPOC-11 by the CD-FOS/glucose/sucrose plate assay. The strains were grown on the CD agar plate supplemented with 1% FOS, glucose, sucrose as substrate at 30 ℃ for 72 h. The strains *A. niger* ATCC 20611 and *A. niger ∆fopA* were used as the control. B. The colony diameter of the transformant DPOC-11. The strain *A. niger* ATCC 20611 and *A. niger ∆fopA* were used as the control. Error bars represent the standard deviation of three independent experiments.


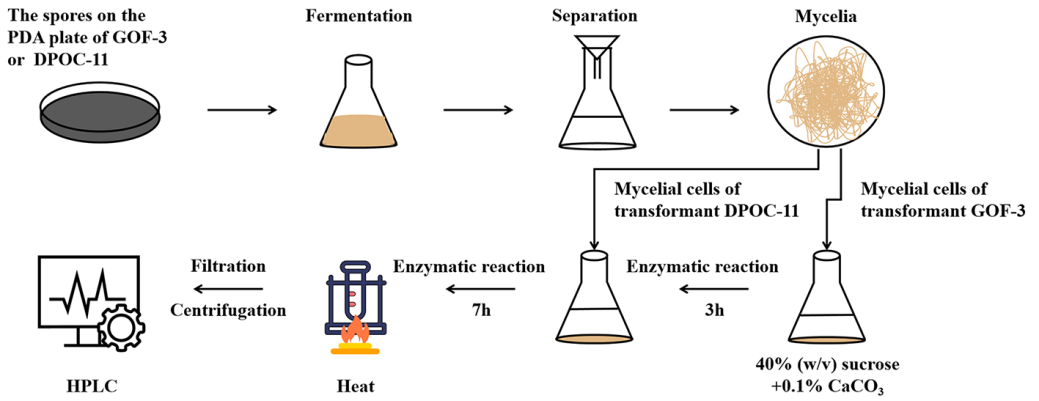


**Fig. S4.** Schematic diagram of the process of FOS synthesis. To collect spores, the strains were cultured on potato dextrose agar medium and grown for 7 d at 30 ℃. The spores were cultured in liquid MM medium and grown for 48 h at 30 ℃. Mycelia were collected and used directly for FOS synthesis. Firstly, the reaction mixture consisting of the collected mycelial cells of *A. niger* GOF-3 (6 units of enzyme per g sucrose), 40% (w/v) sucrose, 0.1% CaCO_3_ in 50 mM citrate phosphate buffer (pH 5.5) was incubated on a shaker at 40 ℃ for 3 h. Secondly, the collected mycelial cells of *A. niger* DPOC-11 were added to the FOS synthesis reaction system. After 7 h, the mixture was taken at the appropriate time and heated to inactivate enzyme. Then the supernatant was collected by filtration. Finally, the concentration of sample was analyzed by a high-performance liquid chromatography system (HPLC).
